# Expression of the inflammatory chemokines CCL5, CCL3 and CXCL10 in juvenile idiopathic arthritis, and demonstration of CCL5 production by an atypical subset of CD8+ T cells

**DOI:** 10.1186/ar1913

**Published:** 2006-02-28

**Authors:** Daniel S Pharoah, Hemlata Varsani, Richard W Tatham, Katy R Newton, Wilco de Jager, Berent J Prakken, Nigel Klein, Lucy R Wedderburn

**Affiliations:** 1 Rheumatology Unit, Institute of Child Health, UCL, London, UK; 2 Department of Paediatric Immunology, Wilhelmina Children's Hospital, University Medical Centre, Utrecht; 3 Microbiology/Infectious Disease Unit, Institute of Child Health, UCL, London, UK

## Abstract

This study focuses upon three chemokines, namely CCL5, CXCL10 and CCL3, which are potential novel therapeutic targets in arthritis. The aim of the study was to analyse the expression and production of these three chemokines within the joints of children with juvenile idiopathic arthritis (JIA) of the oligoarticular and polyarticular subtypes. All three of these chemokines are highly expressed at the level of mRNA, with the most significant increase in mRNA levels being demonstrated for CCL5 when compared with matched peripheral blood samples and controls. We show that high levels of all three chemokines are present in synovial fluid of children with JIA. We investigate the major source of CCL5 from inflammatory synovial cells, which we show to be CD8+ T cells. This CD8+ synovial T cell population has an unexpected phenotype that has not been described previously, being CCR7- yet predominantly CD28+ and CD45RA-. These cells contain high levels of stored intracellular CCL5, and rapid release of CCL5 takes place on T cell stimulation, without requiring new protein synthesis. In addition, we demonstrate that CCL5 is present in synovial biopsies from these patients, in particular on the endothelium of small and medium sized vessels. We believe this to be the first in depth analysis of these mediators of inflammation in JIA.

## Introduction

The hyperplastic and highly vascular synovial tissue that characterises the synovitis of juvenile idiopathic arthritis (JIA) has a dense infiltrate of activated inflammatory T cells, as well as B cells, macrophages and dendritic cells [1-3]. To enter the inflamed site, these cells migrate across an endothelial barrier, a complex process that involves molecular interactions between several receptor-ligand pairs [4,5]. Chemokines are small secreted chemo-attractant molecules involved in such leukocyte trafficking, as well as playing important roles in lymphoid homeostasis and development [6-8]. Functionally distinct subsets of leukocytes express different chemokine receptors: thus, recently activated, effector and memory T cells express high levels of the receptors that bind inflammatory chemokines, thought to facilitate their accumulation at inflammatory sites, compared to naïve cells. Similarly, chemokine receptor expression can be used to distinguish Th-1 T cells (which typically express CXCR3 and CCR5) from Th-2 populations (typically CCR3 positive) [9-11], or 'central' from 'effector' memory T cell populations [12].

As well as mediating chemoattraction, chemokines may also play a direct role in the activation of leukocytes. For example, the chemokine CCL5 (also known as 'regulated upon activation, normally T cell expressed and secreted' (RANTES)) activates T cells when in high concentration through a tyrosine kinase pathway [13,14], leads to production of IFNγ by T cells [15] and may induce maturation of dendritic cells [16]. Thus, migration of T cells under a chemokine gradient into an inflamed site such as the joint in JIA may itself lead to further T cell activation. Furthermore, several of the inflammatory chemokines have recently been shown to be able to increase T cell activation during T cell-antigen presenting cell interaction through their recruitment to the immunological synapse [17].

We have previously shown that inflammatory T cells in the joint in JIA are predominantly of an activated memory phenotype and express high levels of the chemokine receptors CCR5 and CXCR3, and that this correlates with the highly Th-1 skewed phenotype of synovial T cells, which make high levels of IFNγ [18]. A recent study has extended these data by showing that the CCR5+IFNγ+CD4+ synovial cells were enriched within the CCR7- effector memory population, while CXCR3 was also highly expressed in CCR7+ cells, and that these two receptors may be differentially expressed in different areas of synovial tissue [19].

A reduction in T cell migration to the joint in rheumatoid arthritis (RA) has been observed after treatment with anti-tumour necrosis factor therapy or cyclophosphamide [20-22], and the number of peripheral blood T cells expressing CXCR3 has been shown to rise after anti-tumour necrosis factor therapy for RA, an observation that may be explained by reduced recruitment to the joint [23]. A recent phase 1b trial of CCR1 blockade in RA showed clinical benefit at 15 days in those treated with a CCR1 antagonist compared to controls, and a significant decrease in cellularity in synovial biopsies was seen in the treated group [24]. Thus, chemokines and their receptors represent potential targets for new therapeutics [25,26] and drugs that block chemokine-mediated processes might provide synergy with the cytokine blocking biological agents that are now available.

In animal models of arthritis and inflammation, some chemokine blocking agents have been shown to ameliorate or inhibit disease. Thus, antibody to block RANTES inhibited adjuvant-induced arthritis in rats, [27] and anti-CXCR3 antibody can block inflammation in a mouse model of peritonitis [28]. The amino-terminal methionylated RANTES antagonist, met-RANTES, has been shown to block disease in both collagen-induced arthritis and recently adjuvant-induced arthritis [29,30]. Thus, evidence for the use of chemokine blockade is encouraging. For some chemokine receptors expressed on inflammatory cells, however, data from animal models have provided conflicting results. Blockade of CCR2 in collagen-induced arthritis produced varying results, with the effect being critically dependent on the timing of blockade, suggesting that in the late phase of disease, other populations of cells, perhaps with a regulatory function, may express CCR2 [31]. Therefore, to design and direct therapies based upon chemokine blockade accurately, it is important to understand the relative contribution of the various chemokines to inflammation, as well as the triggers for, and sites of, their production in human arthritis.

In this study, we have investigated the expression in JIA patients of three of the ligands for the receptors CCR5 or CXCR3. We demonstrate that the chemokines CCL5 (RANTES) and CCL3 (also known as macrophage inflammatory protein (MIP)-1α), ligands for CCR5, and CXCL10 (also known as IFNγ-induced protein (IP)-10), a ligand for CXCR3, are expressed in the inflamed joint in JIA at higher levels than in peripheral blood. High levels of CCL5 protein is demonstrated in synovial CD8+ T cells, from which it is rapidly released on T cell receptor triggering, a response that does not require new protein synthesis. Our data suggest that the chemokines under investigation here are differentially regulated in the inflamed joint compared to healthy tissues. Inhibition of chemokine release, or blockade of their action, may be important pathways to consider in the search for novel therapies to block inflammation in JIA.

## Materials and methods

### Patients and samples

This study was performed on samples from 50 children (33 females, 17 males) with JIA who met the International League Against Rheumatism (ILAR) criteria [32], 5 healthy control adults, and 14 healthy control children. All the patients attended Great Ormond Street Hospital, London. The study had approval from the ethical review committee (LREC) of Great Ormond Street Hospital and the Institute of Child Health. Full informed consent was obtained from parents of each child in the study. Paired samples of peripheral blood (PB) and synovial fluid (SF) were obtained at the time of clinically indicated arthrocentesis. All samples were processed within one hour of removal from the patient. PB mononuclear cells (PBMCs) were isolated by standard Ficoll-Hypaque density centrifugation. For the preparation of SF mononuclear cells (SFMCs), samples were first treated with Hyaluronidase (Sigma, Poole, Dorset, UK) 10 U/ml for 30 minutes at 37°C before density gradient isolation. In some experiments, cells were separated into cells adherent to plastic and non-adherent cells by incubation at 37°C for 60 minutes. For a subset of samples, T cells were purified from PBMCs or SFMCs by negative selection using monoclonal antibodies to CD14 (UCHM1), CD19 (BU12) (generous gifts from Professor P Beverley), CD16 (BL-LGL/1; Sigma) and CD13 (WM15; Pharmingen Oxford UK) followed by anti-mouse IgG magnetic beads (Miltenyi Biotech Bisley Surrey UK) according to standard methods. This routinely yielded CD3^+ ^cells at a purity of 92% to 96%. In parallel with the cell preparations, small volumes of PB or SF were used to prepare cell-free fluid (plasma or synovial fluid, respectively) using a protocol to minimise platelet release to prevent release of CCL5 from platelets [33]. These samples were snap frozen at -80°C within 1 hour.

### Analysis of mRNA levels of chemokines

Total RNA was isolated from 2 × 10^6 ^cells (PBMC, SFMC or separated cell fractions as indicated) using RNAzol (Biogenesis, Poole, UK) according to the manufactuer's instructions. RNA (2 to 5 μg) was used to generate cDNA using oligo dT (Boehringer Manheim, Lewes Sussex, UK) and Superscript II reverse transcriptase (Gibco, Paisley, UK). Primers for RT-PCR (each written 5'-3') were: CCL5, forward CCATGAAGGTCTCCGCGGCAC, reverse CCTAGCTCATCTCCAAAGAG; CCL3, forward ATGCAGGTCTCCACTGCTGC, reverse TCAGGCACTCAGCTCCAGGTC; CXCL10, forward AAGGATGGACCACACAGAGG, reverse ACCCTTGGAAGATGGGAAAG. Control primers for human β-actin were forward ATGGATGATGATATCGCC, reverse ATCTTCTCGCGGTTGGCCTT. PCR reactions were performed using 1/60th of each cDNA and products visualised on an ethidium-stained 1.5% agarose gel.

In some experiments, PCR products were blotted onto nitrocellulose (Hybond N+, Amersham, Little Chalfont, Bucks, UK) by standard methods and membranes probed using ^32^P-labelled specific oligonucleotides. Probe sequences, designed using Biowire Jellyfish Software (LabVelocity, San Francisco, CA, USA), were: β-actin, AGAAAATCTGGCACCACACC; CCL5, AACCCAGCAGTCGTCTTTGT. Densitometry analysis was performed using the Bio-Rad FX imager (Bio-Rad Laboratories, Hercules, CA, USA) and QuantityOne software (Bio-Rad Laboratories). Chemokine band densities were normalised against β-actin for the same cDNA by dividing the chemokine signal by the actin signal and multiplying by 100.

### Measurement of chemokines in synovial fluid by multiplex immunoassay

Levels of CCL5, CCL3 and CXCL10 were measured in plasma and SF samples after only one thaw. Heterophilic immunoglobulins were pre-absorbed from samples with protein-L and the multiplex immunoassay for chemokines carried out as described [34,35]. Samples were run undiluted and diluted 1:50. Values of blanks were subtracted from all readings. Measurements and data analysis of all assays were performed using the Bio-Plex system in combination with the Bio-Plex Manager software version 3.0 using five parametric curve fitting (Bio-Rad Laboratories).

### Cell culture, ELISA and flow cytometry

SFMCs and PBMCs were cultured overnight at a concentration of 1 × 10^6 ^cells/ml in RPMI/10% fetal calf serum, either alone, or in the presence of the ER blocking agent Brefeldin A (Sigma) at 5 μg/ml for the final 4 hours of culture. In some experiments, T cells purified by negative selection as above were cultured for 6 hours in wells precoated with antibodies to CD3 (UCHT1) and CD28 (CD28.2 clone), each coated at 5 μg/ml in the presence or absence of cyclohexamide (10 μg/ml). Supernatants from these cultures were assayed in duplicate for CCL5 by ELISA (Quantikine, R&D Systems, Abingdon, Oxford, UK) according to the manufacturer's instructions.

Standard three colour flow cytometry was performed for surface proteins using anti-human CD3-PE (BD Pharmingen) anti-CD8-FITC or anti-CD8-QR (Sigma), anti-CD28-FITC or anti-CD28-PE (BD Pharmingen), anti-CD45RA-PE (Serotec, Kidlington, Oxford, UK) and anti-CCR7 (R&D Systems). For intracellular staining of CCL5 chemokine, cells were fixed in 4% paraformaldehyde (Sigma) in phosphate-buffered saline and permeabilised in 0.1% saponin; antibodies and wash buffer for intracellular staining also contained 1% saponin. Anti-human CCL5-FITC antibody was from R&D. FACS data were collected on a FACScan (Becton Dickinson, Moutain View, CA, USA) using Cellquest software (Becton Dickinson, Moutain View, CA, USA); 20,000 to 50,000 events were collected for each condition and cells gated by scatter properties.

### Immunohistochemical staining of synovial tissue

Synovial tissue was collected at the time of synovial biopsy or therapeutically indicated arthroplasty and snap frozen until further use. Seven micron sections were cut, fixed in acetone and stained by standard immunohistochemistry methods using murine anti human-CCL5 (Biosource, Camarillo CA USA) or matched isotype control (Becton Dickinson) followed by donkey anti-mouse-Ig and standard avidin biotin complex protocol.

### Statistics

Data were analysed using SSPS V11 (Chicago, Illinois USA) for analysis of continuous variables (age, disease duration) between disease subtypes. For non-continuous variables (sex, RF, anti-nuclear antibody (ANA) and drug status), Fisher's Exact test was used and a level of 0.05 taken as significant. Where few patients of any subtype were positive for a specific feature, such as use of oral prednisolone, or children not on non-steroidal anti-inflammatory drugs, these data were not formally analysed due to the very small numbers. For comparison of measurements from blood and SF for sets of patients, data were first analysed to confirm normal distribution and then compared by a paired students *t *test. For comparison of chemokines measured in different patient sets, data were first analysed to confirm normal distribution and then compared by unpaired *t *tests.

## Results

Paired samples of SF and blood from a total of 50 children with JIA (21 persistent oligoarticular, 16 extended oligoarticular, and 13 polyarticular) were analysed in this study. In addition, plasma from 14 healthy control children (6 female, 8 male; mean age 7.55 years, standard deviation (SD) ± 2.59 years) and PBMCs from 5 healthy control adults (4 female, 1 male; mean age 29.74 years, SD ± 2.77) were included. The disease characteristics of the 50 JIA patients are shown in Table 1. The mean age of the JIA patients at time of sampling was 10.60 years (SD ± 4.72 years) and mean duration of disease was 5.48 years (SD ± 4.95 years). As expected, the oligoarticular groups showed a trend for younger age and shorter disease duration at sampling; however, statistical analysis showed no significant differences in these variables. Some differences between the subtypes in clinical features were seen, however, as expected. The presence of ANA was significantly lower in the polyarticular group compared to the persistent oligoarticular (*p* = 0.001) and extended oligoarticular (*p* = 0.02) groups, and the use of methotrexate (MTX) was significantly lower in the persistent oligoarticular group compared to the extended oligoarticular (*p* = 0.02) and polyarticular (*p* = 0.007) groups.

### Increased transcription of inflammatory chemokines in synovial fluid cells

**Figure 1:**
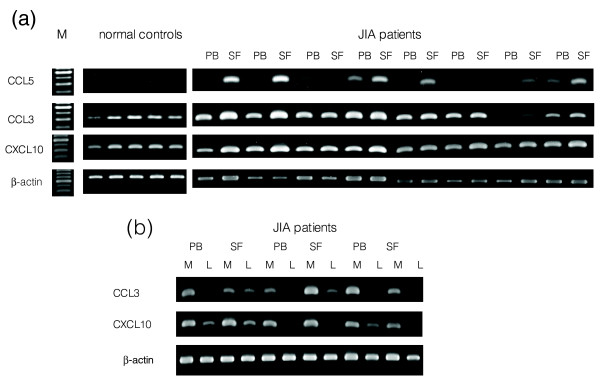
Expression of mRNA for three inflammatory chemokines in juvenile idiopathic arthritis. **(a) **Amplification products of mRNA for CCL5, CCL3 and CXCL10 after RT-PCR from peripheral blood (PB) and synovial fluid (SF) mononuclear cells from patients (*n* = 8) with juvenile idiopathic arthritis (JIA; 5 persistent oligoarticular, 3 extended oligoarticular) and 5 controls. Amplification of β-actin acted as control. **(b) **Amplification of mRNA for CCL3, CXCL10 and β-actin from myeloid (M) or lymphoid (L) cells purified from three representative JIA patients (one persistent oligoarticular, one extended oligoarticular, and one polyarticular).

Levels of mRNA of the three inflammatory chemokines CCL5 (RANTES), CCL3 (MIP-1α) and CXCL10 (IP-10) were measured by RT-PCR in eight pairs of PBMCs or SFMCs from patients with JIA (five persistent oligoarticular, three extended oligoarticular), and PBMCs from five healthy controls (Figure 1a), using equal cell numbers for each cDNA preparation. Constitutive transcription of CXCL10 was demonstrated in all PBMCs, while CCL3 mRNA was readily detected in control PBMCs and seven of eight PBMCs from JIA patients. mRNA for CXCL10 and CCL3 were readily detected in all SFMC samples. CCL5 was not detected in control PBMCs and was absent or only weakly detectable in patient PBMCs. In contrast, mRNA for CCL5 was present in all eight SFMC samples and at high levels in five out of eight of these (Figure 1a).

**Figure 2:**
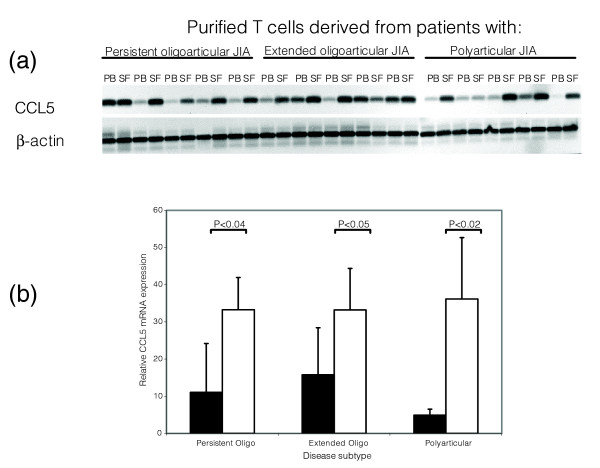
High expression of mRNA for CCL5 in purified T cells from the joint of patients with juvenile idiopathic arthritis (JIA). **(a) **Oligonucleotide-specific probing of blotted amplification products of CCL5 after RT-PCR from paired sets of purified T cells from 15 JIA patients (5 persistent oligoarticular, 5 extended oligoarticular, 5 polyarticular). **(b) **Relative levels of CCL5 mRNA in purified T cells from each JIA subgroup. Black bars, peripheral blood (PB) CD3+ cells; white bars, synovial fluid (SF) CD3+ cells. Bars represent mean values; error bars one standard deviation.

After separation into myeloid and lymphoid populations, RT-PCR of CCL3 and CXCL10 on separated cells from three JIA patients (one persistent oligoarticular, one extended oligoarticular, and one polyarticular) showed that these two chemokines were transcribed predominantly in the myeloid population (Figure 1b) as also seen in controls (data not shown). In contrast, cells expressing highest levels of CCL5 were in the non-adherent, predominantly lymphocyte population. To further quantify transcription of these chemokines in synovial lymphocytes, purified CD3+ cells (2 × 10^6^) from PBMCs and SFMCs from 15 JIA patients (5 persistent oligoarticular, 5 extended oligoarticular, 5 polyarticular) were used to prepare cDNA for semi-quantitative RT-PCR. Amplification products were blotted and probed with a specific primer (Figure 2a) and results quantified by densitometry. The quantity of CCL5 mRNA was expressed as a ratio of expression of β-actin. CCL5 mRNA levels were significantly increased in synovial T cells compared to peripheral blood T cells in all three disease subtypes (Figure 2b). Interestingly the greatest fold increase of CCL5 mRNA levels in synovial T cells compared to matched peripheral blood was seen in those children with polyarticular JIA (Figure 2b). For CCL3 and CXCL10, although both were expressed at higher levels in myeloid cells than lymphocytes, semi-quantitative analysis of these two transcripts in purified synovial fluid and peripheral blood T cells also showed that synovial T cells expressed higher levels of these two chemokines (data not shown).

When these PCR data for each chemokine were analysed according to drug use at time of sampling, there were no significant differences in the levels of the three mRNA species in patients on MTX compared to those not on MTX (data not shown).

### Production of CCL5 by synovial fluid T cells

**Figure 3:**
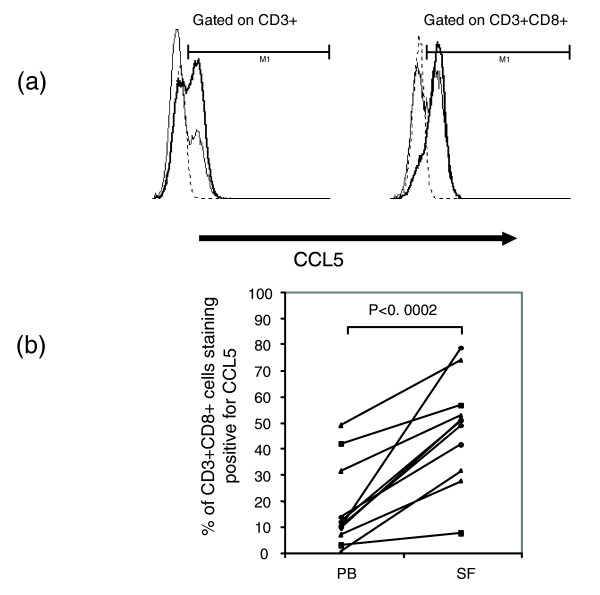
Intracellular flow cytometric analysis of CCL5+ synovial T cells. Peripheral blood (PB) and synovial cells were stained for CD3, CD8 and CCL5. **(a) **Histograms represent CCL5 expression in cells from PB (thin line), or synovial fluid (SF; bold line), compared to staining by isotype control (dashed line), with events gated either on all CD3+ cells (left panel), or CD3+CD8+ cells only (right panel). Marker (M1) indicates CCL5 positive cells. **(b) **Summary of flow cytometric analysis of CCL5 expression from 11 JIA patients (4 persistent oligoarticular, closed triangles; 5 extended oligoarticular, closed circles; and 2 polyarticular, closed squares), indicating number of CD3+CD8+ cells staining positive for CCL5 in PB and SF.

To investigate the differences in mRNA production of CCL5 seen between peripheral blood and synovial T cells, we further investigated the population producing CCL5 from the joints of patients with JIA by flow cytometric analysis. CCL5 protein was detected within T lymphocytes from both blood and synovial fluid without any stimulation, and these were shown to be predominantly CD8+ cells in both compartments (Figure 3a). In all 11 JIA patients tested (4 persistent oligoarticular, 5 extended oligoarticular, 2 polyarticular), the number of CD8+T cells staining positive for RANTES was considerably higher in synovial fluid T cells than in peripheral blood T cells and the difference between the two (PB compared to SF) was statistically significant (*p* < 0.0002; Figure 3b). The addition of Brefeldin A or monensin to unstimulated cultures did not increase the amount of CCL5 detected (data not shown). This correlates with recent reports that CCL5 secretion from memory and effector CD8+ T cells is from stored granules, thought to be distinct from lysosomal secretory granules, and that this secretion of CCL5 is resistant to ER and Golgi blockade [36,37].

**Figure 4:**
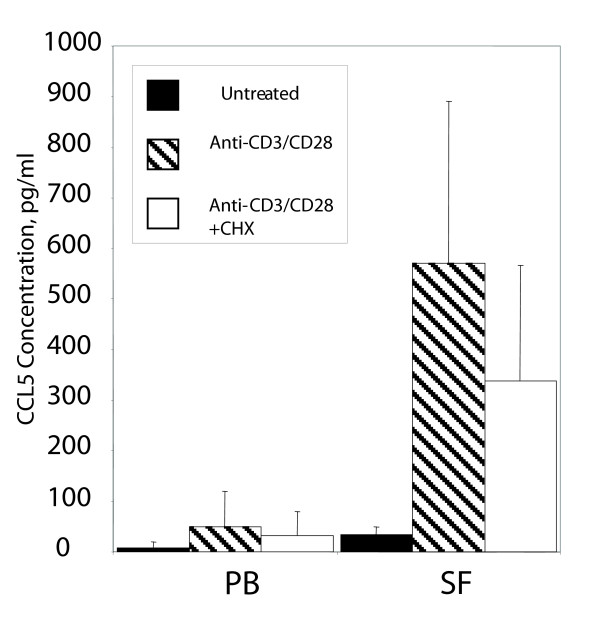
Release of CCL5 from synovial cells is very rapid and largely independent of new protein synthesis. Purified T cells from peripheral blood (PB) and synovial fluid (SF) were stimulated with anti-CD3 and anti-CD28, with (clear bars) or without (hatched bars) cyclohexamide (to block protein synthesis), or in control medium (black bars). Supernatants were assayed by standard ELISA for CCL5. Bars represent mean values of CCL5, and error bars one standard deviation.

To demonstrate the active secretion of this stored CCL5, we stimulated purified T cells from blood and synovial fluid (*n* = 4) for 6 hours using anti-CD3 and anti-CD28. As expected, PB T cells showed low levels of secreted CCL5 without stimulation, and responded to stimulation by an increased release of CCL5 that was partially inhibited (33% inhibition compared to maximal release) by cyclohexamide (Figure 4). Synovial fluid T cells showed higher basal levels of CCL5 release in this assay, and a greatly increased release of the chemokine upon stimulation, which was also only partially blocked (40% inhibition compared to maximal release) by inhibition of new protein synthesis (Figure 4).

### Phenotype of CCL5+ T cells within the joint

**Figure 5:**
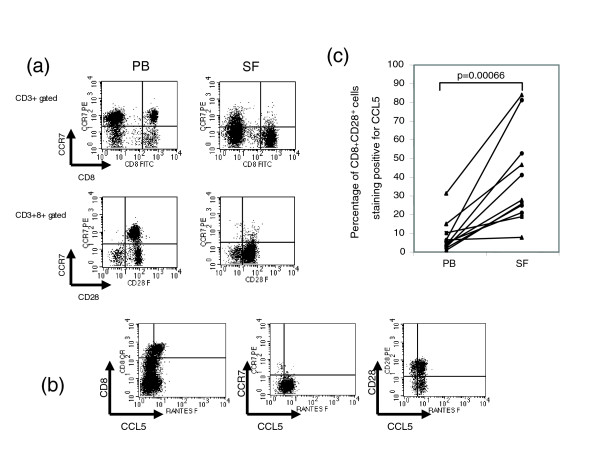
The phenotype of CCL5+ synovial T cells diverges from the effector memory phenotype. Flow cytometric analysis of peripheral blood (PB) and synovial cells. **(a) **Cells were stained for CD3, CD8 and CR7 and CD28. The majority of synovial fluid (SF) T cells were CCR7- (upper panels). However, within the CCR7- population of CD8+T cells, synovial T cells showed maintained high expression of CD28 compared to CD8+CCR7- T cells from PB (lower panel). **(b) **Costaining for CD8, CCR7, CCL5 and CD28 confirmed that the synovial CCR7-CD28+CD8+ cells also contained high levels of intracellular CCL5; positive staining for CCL5 was defined by comparison with isotype-matched control staining. **(c) **The pattern of CCL5+ staining seen in CD8+CD28+ T cells was seen in a total of 11 sets of samples (4 persistent oligoarticular, closed triangles; 5 extended oligoarticular, closed circles; and 2 polyarticular, closed squares), when comparing cells from PB and SF.

CD8+ T cells of the effector and memory compartments have been shown to express high levels of the CCR5 chemokine receptor [38] as well as producing the chemokine CCL5 [39,40]. The memory population may be further divided by the expression or loss of expression of CCR7 [12]. We and others have previously shown that the majority of T cells within the joint, of both CD4 and CD8 populations, express high levels of CCR5 and CD45RO [18,19,41]. It is known, however, that a proportion of memory CD8 T cells, presumed 'revertants', express CD45RA [42-44]. These revertant CD8 T cells are typically of the CCR7-CD28- phenotype. We have previously shown that within the inflamed joint in JIA, a high number of these cells are still CD28+ compared to the conventional memory CD8+ population in blood [3]. We therefore asked whether or not the high number of CCL5+CD8+ T cells had a phenotype typical of 'terminal effector' T cells. We analysed expression of CCR7, CD28 and CD45RA on the CD8 cells from the joint, which were producing CCL5. As expected, an increased number of synovial T cells were CCR7- compared to matched PB T cells (Figure 5a) and the great majority of CCL5+ cells were CCR7- (Figure 5b). However, a significant proportion of CCL5+ cells within the synovial fluid were still CD28+ and CD45RA- (Figure 5b), while in PB of both patients and controls, the majority of CD8+CCL5+ T cells were CD28-CD45RA+. In a set of 11 JIA patients tested (4 persistent oligoarticular, 4 extended oligoarticular and 3 polyarticular), CCL5 expression was measured within the CD8+CD28+ T cells in paired samples of blood and synovial fluid T cells. This showed a significant increase in the CCL5 positive cells within this CD8+CD28+ population in the synovial T cells compared to PB (Figure 5c). In addition, these CD28+ CCL5+ CD8+ cells were also predominantly CD45RA-. Thus, within the CCL5+ CD8+ cells, a mean of 81.8% (SD ± 10.3) were CD45RA- and only 18.3% (SD ± 8.1) were CD45RA+.

### Immunohistochemical analysis of CCL5 in synovial tissue

**Figure 6:**
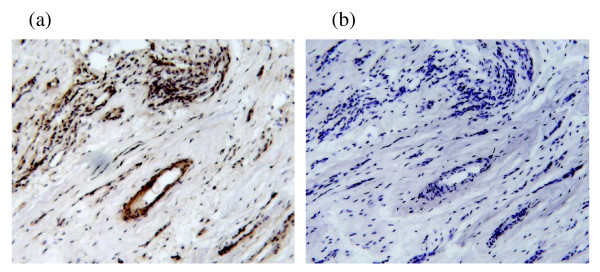
Intense staining for CCL5 on endothelium within inflamed synovial tissue of juvenile idiopathic arthritis patients. Immuno-staining for (a) CCL5 on synovial biopsy tissue taken from a child with oligoarticular juvenile idiopathic arthritis, carried out on a frozen section by standard immunohistochemistry for (b) CCL5 or with isotype control (both images 100×). CCL5 is seen to be expressed on endothelial cells and infiltrating inflammatory cells.

Staining of sections from synovial tissue taken from three patients with JIA showed that many, though not all, of the small and medium sized blood vessels in the highly vascular endothelium expressed CCL5 protein (Figure 6). In addition, staining was demonstrated at the synovial lining and on many infiltrating inflammatory cells.

### Protein levels of inflammatory chemokines in synovial fluid

Protein levels of CCL3 (MIP-α), CXCL10 (IP-10) and CCL5 (RANTES) were measured in plasma and paired SF (prepared by identical method) from a subset of 14 of the patients (4 persistent oligoarticular, 6 extended oligoarticular, 4 polyarticular JIA), and in plasma from 14 healthy control children. All three chemokines were detected in the plasma of both patients and controls, but were significantly higher in the JIA patients than controls (Table 2). Protein levels of CCL3 and CXCL10 were also significantly higher in synovial fluid than paired plasma of patients, with an average increased level of 6.6-fold and 4.9-fold, respectively (Table 2), and were typically in the pg/ml to ng/ml range.

The levels of detectable CCL5 were also measured in SF and plasma. Levels of CCL5 were comparable with previous reports in other inflammatory conditions such as RA, approximately 1 ng/ml [45]. However, levels of free CCL5 in SF were found to be lower than those in plasma in this set of samples (Table 2), with mean plasma levels of 211 ng/ml (Table 2). These data should be interpreted with caution, since platelet release of granules during preparation of samples can artificially raise levels of CCL5 detected by ELISA.

The data were analysed by drug use at the time of sampling, comparing patients on MTX to those not on MTX. Significant differences were seen in the levels of CCL3, which were higher in those on MTX than those not on MTX in both plasma and SF. The mean level of plasma CCL3 in those on MTX was 4,868.67 (SD ± 2,531.37) pg/ml compared to 1,447.97 (SD ± 1,666.38) pg/ml in the non-MTX group (*p* = 0.01), while the mean level of synovial fluid CCL3 in those on MTX was 27,868.88 (SD ± 2,549.33) pg/ml compared to 14,202.63 (SD ± 11,774.61) pg/ml in the non-MTX group (*p* = 0.02). The results of the levels of free chemokines were not analysed separately by disease JIA subtype due to the small numbers in each subgroup.

## Discussion

In this study we have analysed the expression of CCL5, CXCL10 and CCL3 in JIA. We focused upon these three mediators because we have previously shown that receptors for these chemokines are highly expressed on inflammatory T cells from the joint in JIA [18]. mRNA coding for both CCL3 and CXCL10 was readily detected in both the inflammatory cells from the joint (SFMCs) of children with all subtypes of JIA studied and PBMCs from both patients and controls. Both of these chemokines were shown to be predominantly transcribed in cells of myeloid origin from these samples, but no clear differences in mRNA levels were seen between the two compartments in these samples. In contrast, free protein levels of these two chemoattractant mediators were both significantly increased in SF when compared to PB plasma. These data suggest that a gradient exists from blood to the synovial compartment for both CCL3 and CXCL10, which may contribute to the recruitment of inflammatory cells expressing CCR5 and CXCR3, predominantly monocytes/macrophages and memory T cells, to the joint. Our data parallel previous reports of these two chemokines in other forms of arthritis, such as RA, in which levels of CCL3 and CXCL10 measured in SF were higher than those in serum [45,46]. A recent study has shown that synovial T cells from JIA patients do indeed migrate towards a CCL3 gradient [19].

In contrast to CCL3 and CXCL10, levels of transcription of mRNA for CCL5 were clearly increased in synovial cells, predominantly in CD8+ T cells, in all three subtypes of JIA investigated. Synovial CD8+ T cells also contained high levels of the protein CCL5, which was readily detected by intracellular staining, although no increased detection was seen with the use of ER blockade. This correlates with reports that the rapid secretion of CCL5 from memory and effector CD8+ T cells is from stored granules, which are thought to be a unique compartment, distinct from lysosomal secretory granules, and that this secretion is resistant to ER and Golgi blockade [36,37,40]. These CCL5+ T cells also show high expression of the receptor CCR5, such that they may represent a 'positive feedback loop', facilitating their own recruitment.

We analysed the phenotype of the CCL5+CCR5+CD8 T cell population within the joint. Several studies have proposed stages of differentiation for effector and memory CD8 T cells: CD45RA+CD28+CCR7+CCR5- (naïve); CD45RA-CD28+CCR7+CCR5+ (recently activated/memory); CD45RA-CD28-CCR7-CCR5+ (effector memory); and CD45RA+CD28-CCR7-CCR5+ ('terminal effector') [38,47]. However, the exact sequence of phenotypic changes in this differentiation pathway, and whether these differ between anti-viral cytotoxic T lymphocytes (CTL) and cells in chronic inflammatory situations, remains unclear.

The population of CCL5+ T cells within the JIA synovial compartment showed some features of an effector CD8 population (CCR5+CCR7-), but the higher expression of CD28 and low CD45RA expression were discordant with the typical phenotype for a terminally differentiated population. Rather, these cells would appear to fit predominantly into a 'pre-terminal' differentiation state as defined by Champagne and colleagues [47]. The expression of CD28 in the CCL5+ cells may be due to a selective recruitment of activated memory cells that are still expressing CD28, or possibly due to re-expression of CD28 in the joint in the presence of high numbers of cells expressing CD28 ligands such as CD80 or CD86 [48]. Previous evidence suggests that re-expression of CD28 in this population is unlikely. Our results indicate that the usual 'rules' of phenotypic co-expression and regulation in T cell populations may be altered within a chronic inflammatory site, and discordant expression may occur (in this case, in the CD28+CD45RA-CCL5+CCR7- CD8 T cells).

In addition to the high levels of CCL5+ CD8 T cells, which showed rapid release of high levels of this chemokine on T cell receptor triggering, levels of free CCL5 measured in SF samples here were high, compared to previous reports of synovial levels of CCL5 in adult inflammatory arthritis [45,46]. These levels were lower than the free CCL5 levels found in plasma (or serum), where CCL5 levels were present in ng/ml quantities, again paralleling previous reports. Several factors may explain this. It is known that platelet release of CCL5 may occur *in vitro*, for example during clotting or even handling or delay in processing of samples [33]. For this reason, in this study we used plasma samples in which clotting had not occurred and from which platelets were removed with care; synovial samples were treated in a parallel fashion. However, even this protocol may lead to release from platelets, which are far more numerous in blood than in SF, leading to artificially high measurements in plasma. Furthermore, it is known that CCL5 is readily bound by glycosominoglycans and extracellular matrix, which are abundant in inflamed synovium [49]. Chemokines, including CCL5, may also be presented upon, and produced by, endothelial cells, in particular during inflammation [50,51]. In this context, it is interesting that our results from immunohistological analysis of synovial biopsies from JIA showed intense staining of CCL5 protein on vascular endothelium as well as in inflammatory cells. Our results parallel work published in RA biopsy material showing CCL5 staining on both synovial lining blood vessels and perivascular inflammatory cells [52,53]. CCL5, which is 'presented' by endothelium or secreted in the perivascular microenvironment, may contribute significantly to a gradient of CCL5. Thus, it is possible that true levels of bioactive CCL5 within the joint in these patients are higher than those measured in free SF samples. The situation may be further complicated by the presence of other receptors for CCL5, such as the Duffy antigen/receptor for chemokines (DARC) or D6 [51,54,55]. The expression patterns of these chemokine binding proteins and receptors in the different subtypes of JIA remains to be investigated.

## Conclusion

This study has extended our knowledge of the expression of the inflammatory chemokines CCL5, CXCL10 and CCL3 in JIA. All three chemokines were present in SF and, in the case of CCL3 and CXCL10, a large gradient was demonstrated from blood to joint. We have shown that high levels of mRNA and stored protein of CCL5 are present in CD8+ synovial T cells, and that this can be rapidly released without new protein synthesis on stimulation. We have also demonstrated that several of the features of this inflammatory T cell population within the joint, such as the continued high expression of CD28 within an 'effector ' population, are altered compared to normal peripheral blood T cells of this subpopulation. Overall, this study contributes to our understanding of recruitment of T cells to the joint in inflammatory arthritis and suggests that in the microenvironment of the joint, dysregulation of functional patterns of expression may occur.

## Abbreviations

ANA = anti-nuclear antibody; ELISA = enzyme-linked immunosorbent assay; IFN = interferon; IP = IFNγ-induced protein; JIA = juvenile idiopathic arthritis; MC = mononuclear cell; MIP = macrophage inflammatory protein; MTX = methotrexate; PB = peripheral blood; PCR = polymerase chain reaction; RA = rheumatoid arthritis; RANTES = regulated upon activation, normally t-cell expressed and secreted; SD = standard deviation; SF = synovial fluid.

## Competing interests

The authors declare that they have no competing interests.

## Authors' contributions

DP and LRW generated and planned the project. LRW and NK supervised the work. DP, RT and HV generated the PCR and the flow cytometric data. KN assisted with samples and flow cytometry. WdJ and BJP were involved in many discussions and carried out the multiplex assay for chemokine measurement. LRW wrote the manuscript.

## Figures and Tables

**Table 1:** Characteristics of the patients with juvenile idiopathic arthritis included in this study

Characteristic	Disease subtype		
	Persistent oligoarticular (*n *= 21)	Extended oligoarticular (*n *= 16)	Polyarticular (*n *= 13)

Female:male	14:7	11:5	8:5
Mean age at sampling in years (± 1 SD)	9.69 (± 4.71)	9.66 (± 3.18)	13.23 (± 5.51)
Mean disease duration in years (± 1 SD)	3.64 (± 4.70)	6.17 (± 3.92)	7.60 (± 5.21)
Number (%) ANA+	17 (80.9%)	11 (68.8%)	3 (23%)
Number (%) RF positive	0 (0%)	0 (0%)	2 (15%)
Number (%) on MTX at time of sampling	3 (14.3%)	8 (50.0%)	8 (61.5%)
Number (%) on Prednisolone at time of sampling	0 (0%)	2 (12.5%)	1 (7.7%)
Number (%) on NSAID at time of sampling	17 (80.9%)	14 (87.5%)	11 (84.6%)

ANA, anti-nuclear antibody; MTX, methotrexate; NSAID, non-steroidal anti-inflammatory; RF, rheumatoid factor antibody; SD, standard deviation.

**Table 2:** Protein levels of chemokines in plasma and synovial fluid measured in 14 patients with juvenile idiopathic arthritis and 14 age matched healthy control children

Chemokine	Mean levels (pg/ml)				
	Patients (± SD)			Controls (± SD)	

	Plasma	Synovial fluid	*p*^1^	Plasma	*p*^2^

CCL3	3,158 (± 2,618)	21,035 (± 10,829)	<0.0001	55.2 (± 63.2)	<0.0001
CXCL10	1,297 (± 1,148)	6,451 (± 3,174)	<0.0003	87.8 (± 184.9	<0.001
CCL5	211,096 (± 222,730)	743 (± 675)	<0.006	65,402.1 (± 12,962)	<0.05

^1^Comparison of patient plasma to synovial fluid, paired samples. ^2^Comparison of plasma of patients and controls, unpaired samples.
